# Cavitation Resistance, Microstructure, and Surface Topography of Plasma Nitrided Nimonic 80 A Alloy

**DOI:** 10.3390/ma15196654

**Published:** 2022-09-26

**Authors:** Ion Mitelea, Ilare Bordeaşu, Cosmin Belin, Ion-Dragoş Uţu, Corneliu Marius Crăciunescu

**Affiliations:** 1 Department of Materials and Fabrication Engineering, Politehnica University Timisoara, Bulevardul Mihai Viteazul nr.1, 300222 Timisoara, Romania; 2 Department of Mechanical Machines, Equipment and Transports, Politehnica University Timisoara, Bulevardul Mihai Viteazul nr.1, 300222 Timisoara, Romania

**Keywords:** Nimonic alloy, nitriding, cavitation, microstructure

## Abstract

Cavitation erosion of structural materials is a form of wear damage that affects the performance and life of components used in the aerospace, nuclear, and automotive industries, leading to an increase in the frequency of maintenance operations and redesign costs. The cavitation erosion behaviour of the nickel-based superalloy, Nimonic 80 A, was investigated using a piezoceramic crystal vibrator, according to the requirements of ASTM G32-2016. The results showed that plasma nitriding leads to a reduction in the mean erosion penetration depth by approximately ten times and of the erosion rate by the order of six times, compared to the solution heat-treated samples. Typical topographies of cavitation-eroded surfaces show a preferential degradation of the grain boundaries between the γ solid solution phases, of the twins’ boundary, and of the interface between the precipitated particles and the γ solid solution matrix. In the nitrided samples, the cracking initiation is determined by nitride particles, which are hard and brittle. Due to the high mechanical strength of the solid solution γ with the fcc crystal lattice, the appearance of the cavitation surface is uniform, and the fracture has a ductile character.

## 1. Introduction

Ni-based alloys are used in the fabrication of heavy-duty hydraulics and engine hot section components for wear, corrosion, cavitation, and creep in the aerospace, marine, nuclear, petrochemical, and automotive industries [1,2,3]. In analysing the Inconel 690 alloy, Chen and Wu showed that the cavitation erosion damage initiates at the grain boundary, twin boundary, and at the interface between the precipitate and the matrix [4]. Khajuria and Wani [5] studied the wear of Nimonic alloys under dry sliding conditions and concluded that lower wear and friction occurred at high temperature. Sun et al. developed Ni–Fe–Cr based superalloys as a low-cost alternative for hot-corrosion resistance, observing a hardness increase at room temperature, due to the formation of the σ phase [6]. Liu et al. further showed that a diffusion zone in the bonding between single crystal Ni-based and polycrystalline Ni-based superalloys generated a hardness increase due to the precipitation of the fine γ′-strengthening phases [7].

Nickel is known to have a positive effect on the cavitation erosion resistance of steels, [8] and Ni-based alloys are expected to be a possible solution to increase this property. Chen et al. [9] analysed the erosion rate of the Incoloy alloy 865 surface in NaCl solution and found that degradation was caused by the formation of pits and fatigue processes. According to Liu et al. [10], the GH 4738 nickel-based superalloy showed better cavitation erosion resistance than Inconel 601 due to the presence of nanometre-scaled precipitates. Nimonic 80 A, (EN NiCr20TiAl; UNS No. 7080; W. No. 24952 and 24631) has chromium as its main alloying element, which improves the resistance to heat oxidation. The addition of aluminium and titanium further promotes the strengthening of the alloy by ageing. Although engine exhaust valves are affected by cavitation erosion, including those made out of Nimonic 80 A, limited information is available on the solution to overcome this phenomenon. To increase engine efficiency and service life, research efforts are focused on minimising exhaust valve seat wear. Hard coating deposition techniques, such as surface remelting, thermal spraying, PVD evaporation, and ion implantation are frequently used to reduce wear by cavitation [11]. The effects of coating process parameters on cavitation strength have been frequently studied. Navneet et al. [12] studied the cavitation erosion of nickel-based cermet coatings, while Han et al. analysed the same phenomenon for chromium nitride coatings [13].

Nitriding is one of the most important technologies to generate hard surface layers for machine parts and tools. The main reasons for selecting this thermochemical treatment process are:

- the formation of a wear-resistant thin layer, with high hardness (HV = 700–1200 daN/mm^2^);
- the generation of a surface layer with good adhesion to the substrate;
- an increase of long-term fatigue resistance, due to the compression stresses developed in the surface layer;
- improved surface properties to cavitation erosion and corrosion in atmospheric air, water, vapours, etc.;
- higher resistance to soaking up to temperatures of between 500 and 550 °C.

Nitriding proved to be a solution to further improve surface properties based on microstructural modifications [14,15], while the depth of the nitrided layer and the mechanical characteristics of the core can be selected in order to improve the response to contact stresses [16,17].

For Ni-based alloys, Manova et al. [18] observed, for low-energy ion nitriding of the Ni80Cr20 alloy, that a competition exists between nitrogen loss at the surface and the nitrogen supply by the ion beam. Chollet et al. found that the δ, γ′, or γ″ precipitates appear nitrided in similar proportion than the γ matrix [19]. The composition of Nimonic alloys appears to influence the austenite that, according to Eliasen et al. [20], was present in Nimonic 80, 90, and 100 alloys. However, the technical literature is quite scarce in terms of data regarding the cavitation erosion behaviour of the nitrided layers of steels and non-ferrous alloys [13,14,15,16,17]. There are contradictory results related to the effects of nitriding on the cavitation, mainly due to the original microstructure of the nitrided material, due to the presence of the chemical combinations zones and the types of nitrides formed in the surface layer. In this regard, the present paper investigates the response to cavitation erosion of nitrided plasma surfaces of the Nimonic 80A alloy.

## 2. Experimental Procedure

**Figure 1:**
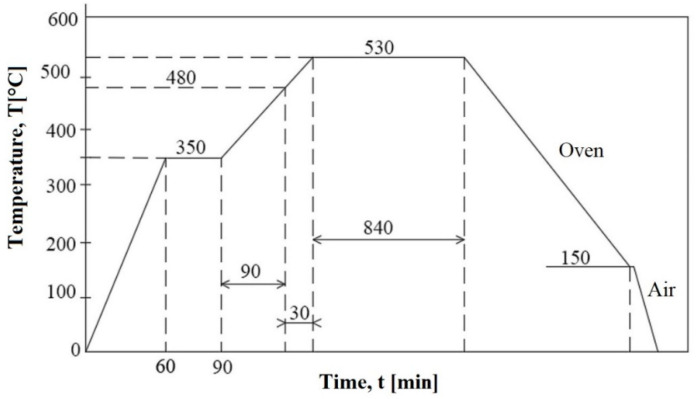
Cyclogram of plasma thermochemical nitriding treatment.

Cavitation samples with the test surface of Φ 15.8 mm were cut out of Nimonic 80 A cylindrical bars (Table 1) and solution-treated at 1080 °C followed by air cooling. Subsequently, one set of samples were subjected to plasma nitriding in a furnace equipped with a PROTHERM 500 device (USA) designed to monitor, control, record, and archive the entire treatment process. The cyclogram of the nitriding thermochemical process is shown in Figure 1.

The samples were placed in the nitriding equipment and preheated to 350 °C for 30 min. This was followed by ammonia addition and further heating to the operating temperature of 530 °C. At 480 ° C, the dissociation of ammonia with the release of active nitrogen atoms began. The holding time at the nitriding temperature (in nitrogen atmosphere) was 840 min. The samples were cooled with the furnace to a temperature of 150 ° C, at which point they were further cooled in air.

Cavitation erosion tests were performed both on samples of the reference material, solution heat-treated, and on samples subsequently subjected to plasma nitriding. The experiments were performed using a piezoceramic crystal vibrator, according to the requirements of ASTM G32-2016 [21]. The parameters used were: 500 W power; 20,000 Hz vibration frequency; 50 μm vibration amplitude; 22 ± 1 °C working fluid temperature [11]. The total duration of each test was 165 min and was divided into 12 intermediate periods (one of 5 and 10 min and 10 of 15 min each). At the beginning and end of each test period, the samples were washed under tap water, distilled water, alcohol, acetone, dried under warm air, and weighed.

Prior to the start of the tests and at the end of each intermediate test period, the surfaces exposed to the cavitation erosion process were examined using a TESCAN 150 Vega 3 LM scanning electron microscope (SEM) to reveal the cavitation damage mechanism. The weighing was performed with a ZAKŁADY analytical balance with 0.01 mg accuracy. The average weight loss (Δm_i_ =$\sum_{j=1}^{3}\frac{\Delta m_{j}}{3}$) was determined for each set of three samples based on the weighted mass losses Δm_1_, Δm_2_, Δm_3_, from each period “i”. The average cumulative mass loss (M_i_ =$\sum_{i=1}^{12}\Delta m_{i}$) was determined over a certain duration of cavitation attack, until completion (165 min). 

According to procedure described elsewhere [22], the measured losses allow the calculation of the cavitation erosion parameters (detailed in Table 2): the mean depth of erosion cumulative penetration MDE_i_, and the corresponding rate of erosion penetration, MDER, which, according to ASTM G32-2016 standards [21], allow for the calculation of MDEmax (maximum depth at the end of the cavitation erosion attack—165 min in the present case) and MDERs (the final value to which the MDER(t) reaches its asymptotic evolution). These parameters allow a good assessment of the surface resistance to cyclic stresses resulting from the impact of the micro-jets generated through the vibrating cavitation mechanism. The evolution of mass loss, in different periods of cavitation attack, is an expression of energy generated by the impact of micro-jets and by the shock waves at the surface of the sample. For the analysis of the mechanical erosion process of the alloy, the experimental points were mediated, with ΔMDEi and MDERi expressing the trend in the material’s behaviour during the cavitation attack.

The mediation of the results, obtained for the cumulative mean depth of erosion (MDE), respectively, the erosion penetration rate (MDER), was achieved using the relations detailed in Table 3 [23]. The analysis of the evolution of the cavitation erosion behaviour and resistance during the accelerated experiments can be achieved using these mediation curves. The assessment is made based on the shape of the curves and the dispersion of the experimental points.

After the cavitation tests measurements, the sample surface and the cross-section through the nitrided layer were metallographically and sclerometrically examined, in order to highlight the way of material degradation from the areas with high nitrogen concentration. The nature of the present phases was identified by X-ray diffraction, and the microstructure and the topography of the eroded surfaces were analysed in a TESCAN Vega 3 LM scanning electron microscope (SEM) (TESCAN Brno, sro, Brno, Czech Republic) equipped with a Bruker Quantax 200 Energy Dispersive X-ray Spectroscopy (EDX) system with a Peltier-cooled XFlash 410M silicone drift detector (Bruker, Billerica, MA, USA). The X-ray diffraction investigation of the samples was performed using a Philips diffractometer (PANalytical X΄Pert Pro Multi-Purpose Diffractometer, Kassel-Waldau, Germany) equipped with a graphite monochromator for Cu-Kα radiation (λ = 1.54 Å) at room temperature. The measurements were performed in 2D geometry, in the range from 0–100°, with a speed of 1 °/min. It was operated at a voltage of 40 KV with a current of 30 mA. Crystallographic identification of the phases in the samples was performed using the JCPDS (Joint Committee on Powder Diffraction Standards) database.

## 3. Results and Discussions

### 3.1. Cavitation Curves

**Figure 2:**
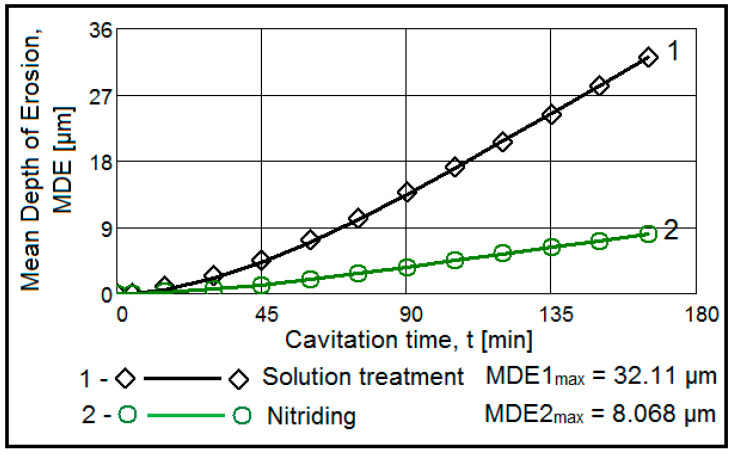
Evolution of mean depth of erosion with the cavitation time (1—solution treatment; 2—solution treatment + nitriding).

**Figure 3:**
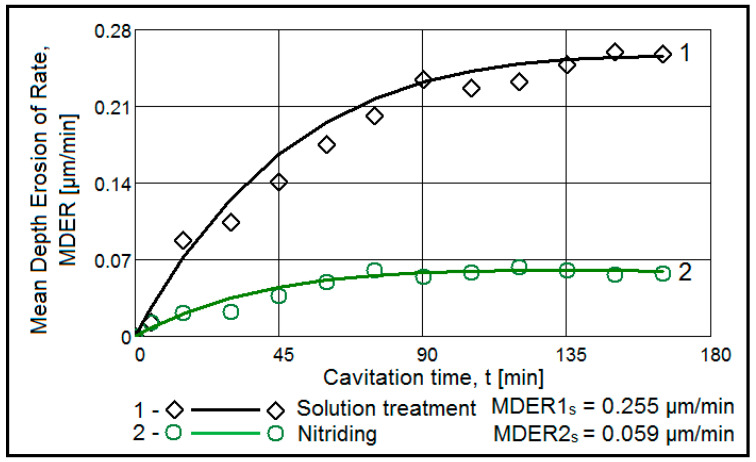
The evolution of mean depth of erosion rate with the cavitation time (1—solution treatment; 2—solution treatment + nitriding).

**Figure 4:**
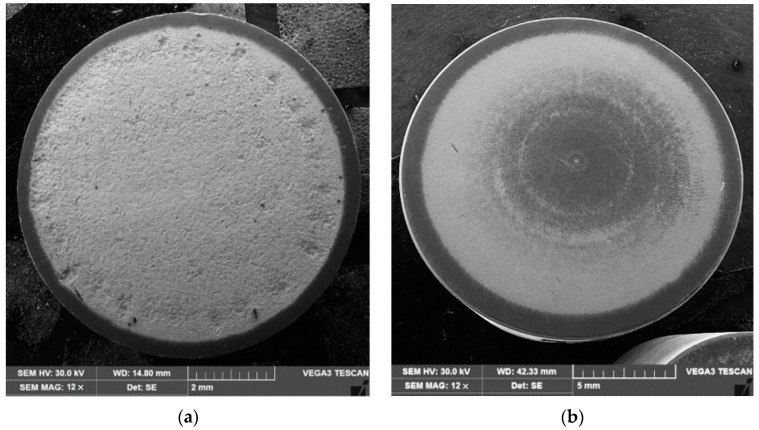
Macrographic images of the surfaces tested for 165 min cavitation time: (**a**) Reference material (solution-treated); (**b**) Solution-treated followed by nitriding.

Figure 2 and Figure 3 show the variation of the MDE(t) and MDER(t) as a function of the duration of the exposure to cavitation erosion, for samples subjected to solution treatment (1080 °C/8 h/air) and for those that were subsequently hardened by plasma nitriding. The following observations result from their analysis:

- in the first 30 min of cavitation, the surface is less affected and withstands the stress induced by the shocks generated by the impact with the cavitation micro-jets. The losses are limited and reflect only the destruction of the roughness left after the technological polishing process at Ra = 0.4 µm. Basically, in this interval, the force of the shocks extends the networks of microcracks associated with the phenomenon of fatigue due to cyclic contractions that manifest during the cavitational cycle. After this period, the sample surface subjected to solution treatment starts to intensely degrade (Figure 4a), while in the nitrided samples, an erosion ring is generated (Figure 4b). The ring is characterised by small craters and caverns of pitting-like shape. It becomes wider and wider and extends to the centre of the specimen. Another aspect observed is the lack of caverns arranged stellar configuration towards the periphery, as can be seen for the samples in the solution treatment state (Figure 4b compared to Figure 4a);
- by using the nitriding thermochemical treatment, the surface resistance to the erosion generated by the microjets developed through the vibrating cavitation increases substantially. Thus, the mean depth of erosion, MDE, (Figure 2), after 165 min of exposure to cavitation and the final constant value (also at 165 min), at which the mean penetration of erosion rate tends to stabilize, MDER, (Figure 3) decrease approximately ten times and six times, respectively;
- the high cavitation resistance of the nitrided layer is confirmed by both the reduced and uniform dispersion of the experimental points towards the mediation curves (very expressive in Figure 3), as well as by the linearization of the MDE(t) curve and the stabilisation of the MDER(t) rate at the maximum value starting with the 60th min. In solution heat-treated samples, this phenomenon starts after 90 min of cavitation.

**Figure 5:**
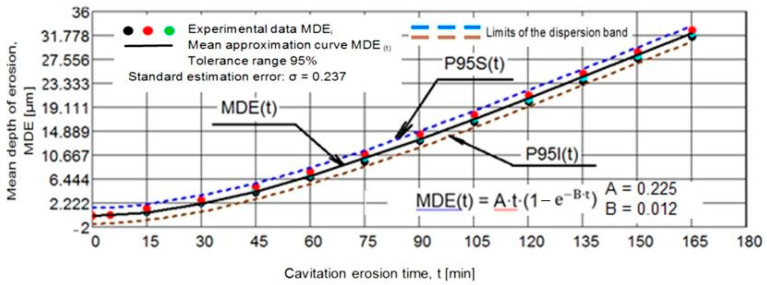
The dispersion range of the experimental values for the samples subjected to solution hardening solution. Statistical reference parameters of the dispersion band.

**Figure 6:**
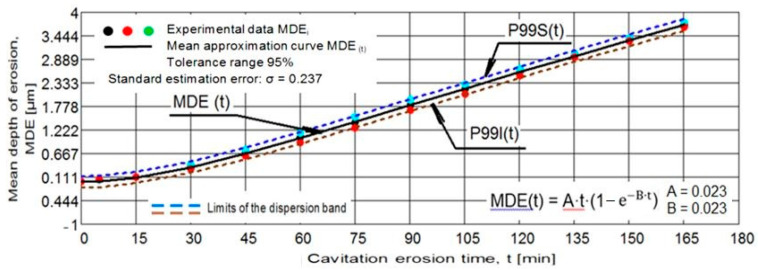
The dispersion range of the experimental values for the nitrided samples. Statistical reference parameters of the dispersion band.

In order to verify the accuracy of the experiment on the three samples of each structural state, the dispersion bands of the experimental values were calculated, with the limits of the tolerance range and the value of the standard estimation error was determined, σ (Figure 5 and Figure 6). The statistical data from the two diagrams (a tolerance range of 95% and a standard estimation error of 0.237) prove the rigorousness of the cavitation testing experiments, in which the functional parameters of the vibrating apparatus that influence the erosive intensity of the process were well controlled. For a process of such complexity as with hydrodynamic and mechanical effects, the differences between the three samples of each state are within normal limits.

### 3.2. Roughness of Cavitation Tested Surfaces

**Figure 7:**
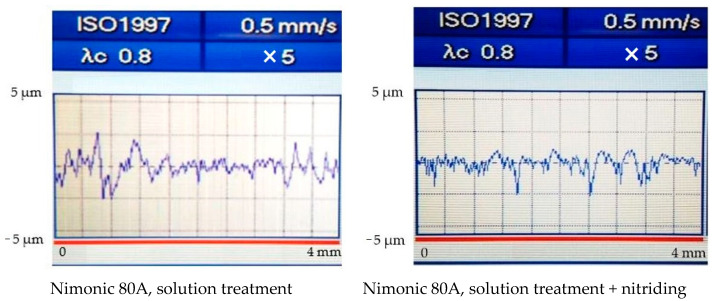
Examples of the surface roughness of the samples subjected to solution treatment (**left**) and nitrided (**right**), tested for cavitation erosion for 165 min.

Roughness measurements of the surface of solution heat-treated samples before and after nitriding and further tested for cavitation erosion for 165 min were made using the Mitutoyo apparatus. For both structural states, the roughness values Ra, Rz, and Rt together with their profilograms, which were recorded in 12–16 randomly chosen areas on the eroded surface from the periphery to the central area, are shown in Figure 7. The comparative analysis of the results highlights large differences between the solution heat-treated samples before and after nitriding treatment. They prove that thermochemical nitriding treatment has a positive effect on improving the cavitation erosion resistance.

Table 4 summarises the comparative values of the roughness parameters and MDE erosion depths, obtained after the cavitation erosion test, for the two treatments applied. The data presented were obtained by arithmetic mediation of the values of the roughness parameters. Their analysis shows that the thermochemical nitriding treatment causes a reduction in the mean penetration depth of erosion, MDE, from 32 µm to 3.27 µm and the main roughness parameter, Rz, from approximately 31 µm la cca. 3 µm.

As well as MDE value, another important cavitation parameter is the erosion rate, MDER. The inverse of this value, towards which it tends to stabilize, MDERs, defines the cavitation resistance, Rcav. Table 5 presents these values compared for both the structural state samples. The data show an increase of up to 532% in the cavitation resistance of the surfaces strengthened by thermochemical nitriding treatment.

### 3.3. Micrographic and X-ray Diffraction Analysis

**Figure 8:**
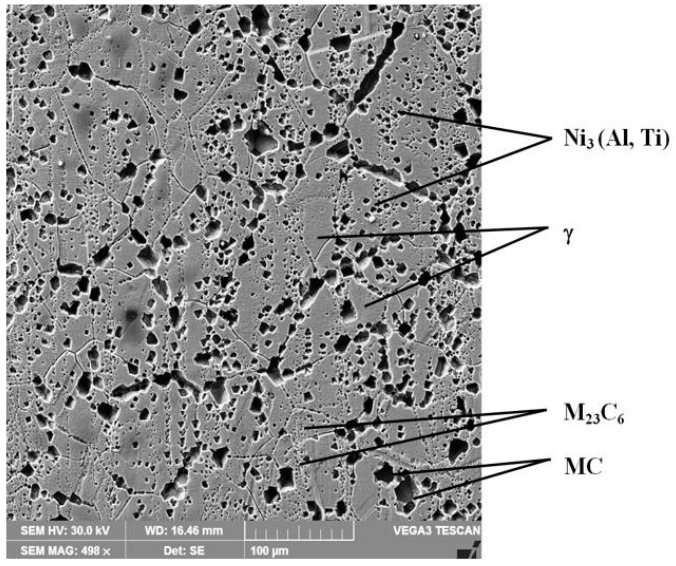
SEM image of the microstructure of solution heat-treated samples.

The SEM micrograph of the reference material, shown in Figure 8, is mainly composed of face-centred cubic (fcc) Ni-based polyhedral grains of solid solution. Present also, with an intergranular presence, are between 20 and 30 µm primary carbides (MC) and M_23_C_6_-type Cr-rich carbides of approximately several hundreds of nm. Intermetallic γ׳ phase precipitates, of Ni_3_(Al, Ti) type, in the range from 40–60 nm, can be observed inside the matrix. These observations are consistent with previously reported research results [6,17,18,19].

**Figure 9:**
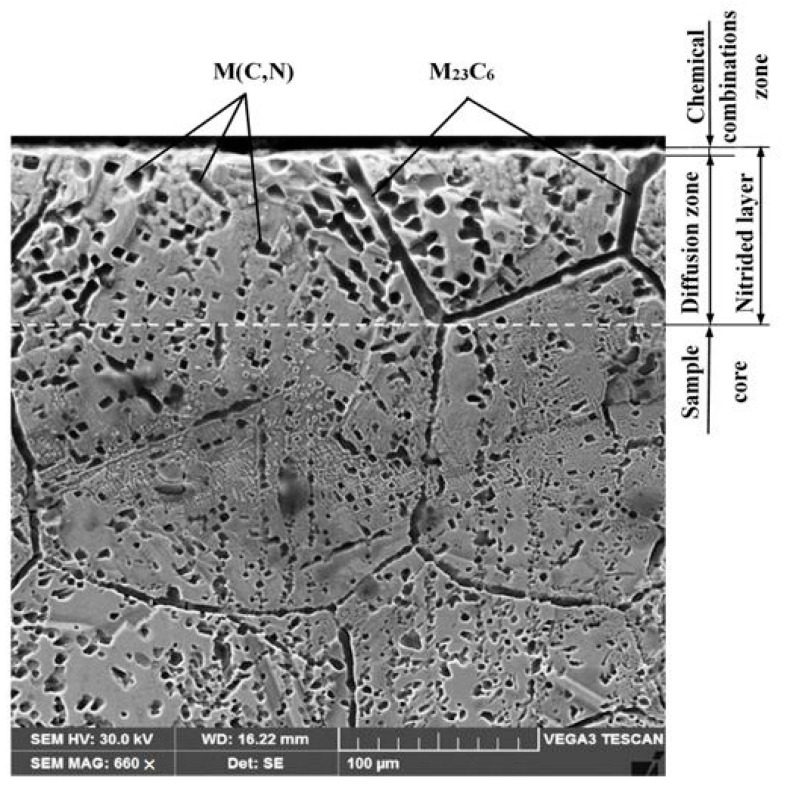
SEM image of the cross-section microstructure of the nitrided layer.

**Figure 10:**
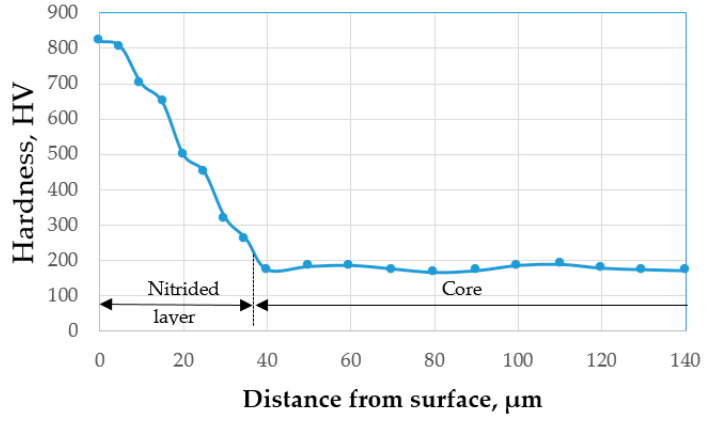
Hardness gradient curve on the cross-section of the nitrided layer.

The SEM image of the cross-section of the nitrided layer (Figure 9), and the hardness gradient analysis, detailed in Figure 10, shows two distinct regions:

- a chemical combinations zone having a light and extremely thin colour, of approximately 3–5 μm; surface hardness had values from 790–820 HV5, more than three times higher than the base material (176–188 HV5);
- a diffusion area of approximately 30–40 μm, consisting of carbonitride particles of the alloying elements (having the size from ≈ 8–10 µm), which are embedded into the solid solution matrix γ.

The nitrides precipitation has drastically reduced the incubation period, allowing the detachment of whole grains, due to the impact of shock waves on the surface. Despite this, after the removal of the first 3–5 μm of the nitride layer, the cavitation erosion resistance was significantly improved.

**Figure 11:**
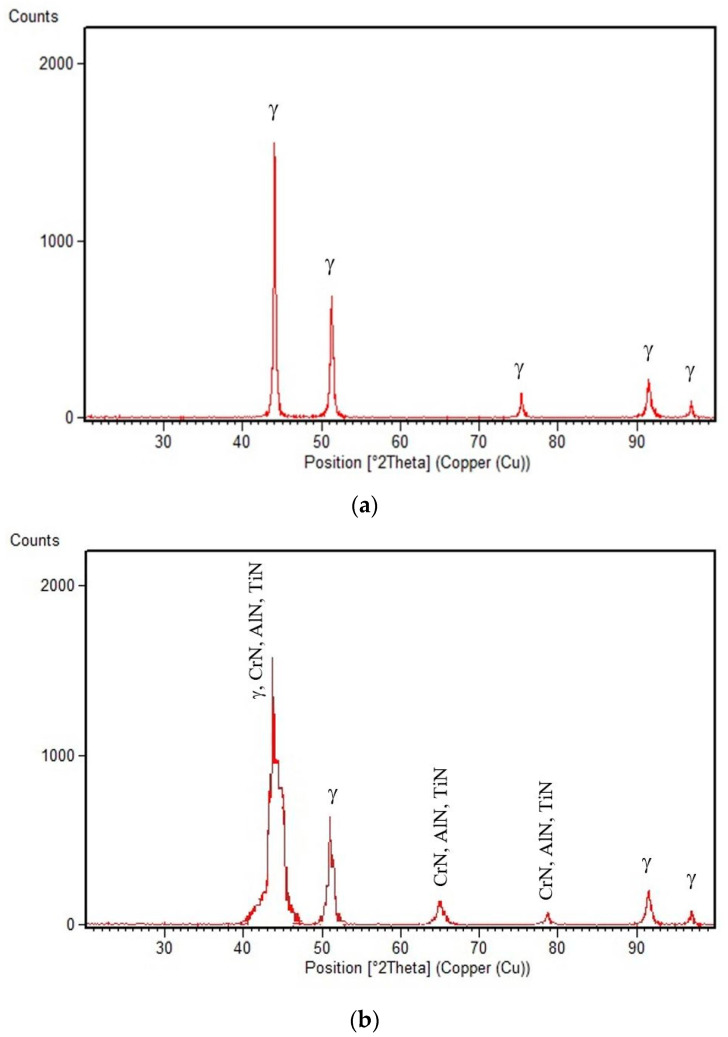
XRD patterns of the samples: (**a**) Reference material; (**b**) Nitrided material.

The X-ray diffractograms recorded for the nitrided surface and for the non-nitrided core are shown in Figure 11. It is observed, that before nitriding, the resulting peaks characterise the solid solution matrix γ (Figure 11a). On the nitrided surface, peaks specific to both the nitride particles of the alloying elements (Cr, Al, Ti) and the solid solution γ were highlighted. TiN and AlN nitrides have crystalline lattice parameters close to those of chromium nitride, CrN. A widening of the solid solution γ peaks is attributed to the formation of Cr, Al, and Ti nitrides, which induce microstresses in the matrix, as well as changes in the chemical composition. Small nitride peaks are also visible (Figure 11b).

### 3.4. Topography of Cavitation Eroded Surfaces

**Figure 12:**
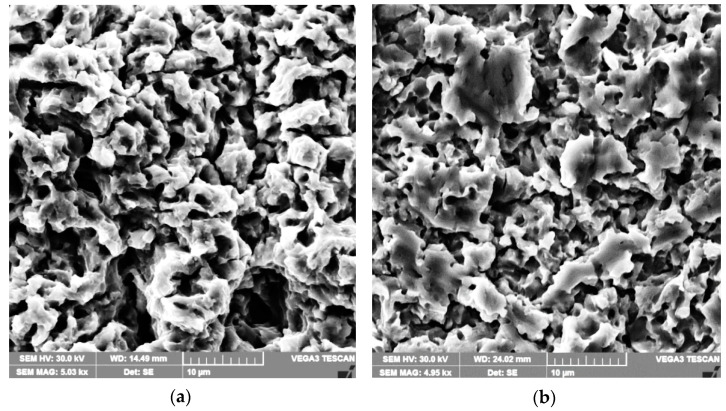
SEM image of the cavitation-eroded sample surfaces: (**a**) Solution treatment; (**b**) Solution treatment followed by nitriding.

The typical topographies of the sample surface for the reference material (Figure 12a) highlight the formation of more than 20 µm deep craters, determined by the preferential formation of cavities at the boundaries between the solid solution γ grains, which are more fragile. It is emphasised that the grain boundaries are less able to absorb the deformation energy due to the stresses induced in the material by cavitation impact waves. As a result, they are subject to faster erosion processes than the microstructure of solid solution γ.

In areas with precipitation in the form of a cellular network, even partial or complete removal of crystalline grains can be observed. In the nitrided state (Figure 12b), compared with the solution-treated state (Figure 12a), the cavitation depths do not exceed 10 µm, and the roughness parameters values decrease by approximately ten times. The microcraters present on the eroded surface are the location of the former nitride particles of the alloying elements.

## 4. Conclusions

Based on the research undertaken within the present scientific work, the following results were obtained:

- the plasma nitriding thermochemical treatment at 530 °C favours an approximately six times reduction in the cavitation erosion rate, respectively, an increase in the cavitation resistance by over 500%, compared to the structural state obtained by solution treatment, 1080 °C/air;
- the microstructure of the nitrided layer consists of an extremely thin area of chemical combinations and a diffusion zone in which the carbonitrides of the alloying elements incorporated in a matrix of solid solution γ with nickel base are present;
- typical topographies of cavitation-eroded surfaces show a preferential degradation of the grain boundaries between the γ solid solution phases, of the twins’ boundary, and of the interface between the precipitated particles and the γ solid solution matrix. In the nitrided samples, the initiation of the cracks is determined by nitride particles, which are hard and brittle;
- due to the high mechanical strength characteristics of the solid solution γ with the fcc crystal lattice, the appearance of the cavitation surface is uniform, and the fracture has a ductile character.

## Figures and Tables

**Table 1:** Chemical composition of Nimonic 80A, weight %.

Chromium	20.5
Titanium	1.98
Aluminum	1.54
Iron	2.14
Cobalt	1.61
Manganese	0.63
Silicon	0.45
Copper	0.12
Carbon	0.08
Sulfur	0.014
Zirconium	0.11
Nickel	Balance

**Table 2:** ASTM G32-2016 parameters for evaluating the resistance and behaviour to cavitation erosion.

Cumulative Mean Depth of Erosion	Erosion Rate
MDE_i_ =$\sum_{i=1}^{12}\Delta MDE_{i}$= $\frac{4\cdot M_{i}}{\rho \cdot \pi \cdot d_{p}^{2}}$ [mm]	MDER_i_ = ΔMDE_i_/Δti
I—represents the test period. Δmi—is the mass of material lost by erosion, in period i, in grams. ρ—alloy density, in grams/mm^3^. Δti—cavitation time corresponding to the period “i” (5 min, 10 min, or 15 min). d_p_—diameter of the sample surface, subjected to cavity attack (d_p_ = 15.8 mm). ΔMDE_i_—the value of the mean erosion penetration depth, achieved by cavitation during the Δti period.	

**Table 3:** Parameters used to evaluate the cumulative mean depth of erosion (MDE), respectively, the erosion penetration rate (MDER).

Mean Penetration Depth of Erosion	Mean Depth of Erosion Rate
MDE(t) = A⋅t⋅(1 − e^−B^^⋅^^t^)	MDER(t) = A⋅(1−e^−B^^⋅^^t^) + A⋅B⋅t⋅e^−B^^⋅^^t^
where: A—is the scale parameter, statistically established for the construction of the approximation/mediation curve, provided that the deviations of the experimental points from it are minimal. B—is the shape parameter of the curve.	

**Table 4:** Comparative values of roughness and MDE parameters (165 min).

Structural Condition	MDE_(165 min.)_ [µm]	Ra [µm]	Rz [µm]	Rt [µm]
Solution treatment	32	5.539	31.598	48.026
Solution treatment + nitriding	3.27	0.465	3.172	4.663

**Table 5:** The effect of nitriding on MDER_s_ and R_cav_ values.

Structural Condition	Cavitation Erosion Resistance Parameter		Variation Compared with Solution Heat-Treated Sample
	MDER_s_ [µm/min]	R_cav_ [min/µm]	
Solution treatment	0.24	4.16	-
Solution treatment + nitriding	0.038	26.32	Increases by 532 % (6.32 times)

## Data Availability

The data reported in this study are available from the authors upon request.
